# Supplementary material for: The interactome of intact mitochondria by cross-linking mass spectrometry provides evidence for coexisting respiratory supercomplexes
Source: Mol Cell Proteomics. 2017 Dec 8;17(2):216–32. doi: 10.1074/mcp.RA117.000470 (PMC5795388; doi:10.1074/mcp.RA117.000470)
Supplement: Supplemental Data [file supp_RA117.000470_133922_0_supp_23978_fzffwf.zip › spectra_annotation/mito_DR_spectra_annotation/88-1-9-1-7-1.pdf]

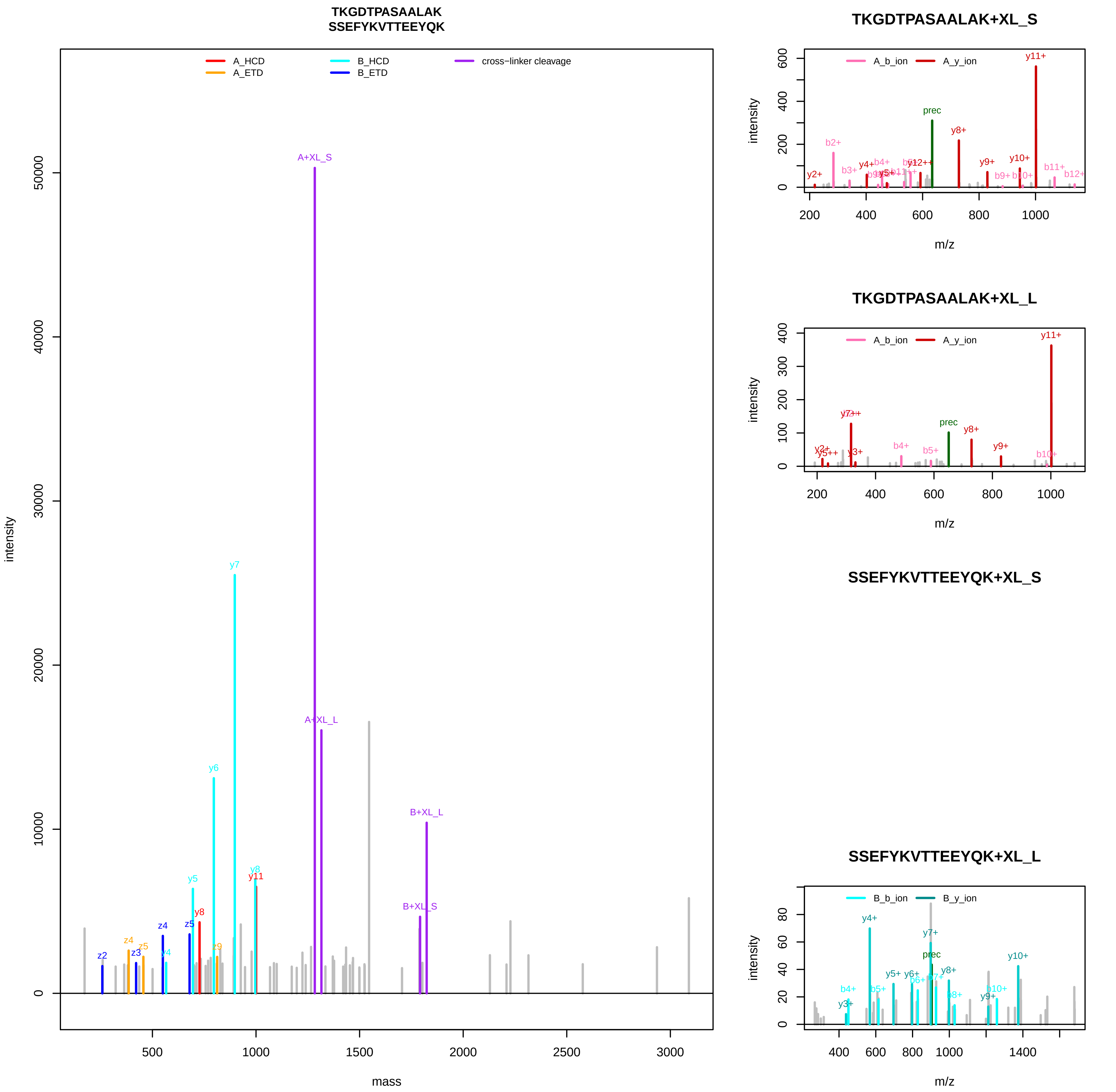
TKGDTPASAALAK
SSEFYKVTTEEYQK
A_HCD
A_ETD
B_HCD
B_ETD
cross−linker cleavage
A+XL_S
A+XL_L
B+XL_L
B+XL_S
y7
y6
y5
y8
y11
y8
y4
z2
z3
z4
z5
z4
z5
z9
intensity
mass
0
10000
20000
30000
40000
50000
500
1000
1500
2000
2500
3000
TKGDTPASAALAK+XL_S
A_b_ion
A_y_ion
prec
y11+
y8+
y9+
y10+
y2+
b2+
b3+
y4+
b4+
b11+
b12+
b9+
b10+
intensity
m/z
0
200
400
600
200
400
600
800
1000
TKGDTPASAALAK+XL_L
A_b_ion
A_y_ion
y11+
prec
y8+
y9+
y2+
y5++
y3+
b4+
b5+
b10+
intensity
m/z
0
100
200
300
400
200
400
600
800
1000
SSEFYKVTTEEYQK+XL_S
SSEFYKVTTEEYQK+XL_L
B_b_ion
B_y_ion
y4+
y7+
prec
y5+
y6+
y8+
y10+
y3+
y9+
b4+
b5+
b6+
b7+
b8+
b10+
intensity
m/z
0
20
40
60
80
400
600
800
1000
1400
